# Continuous paravertebral block for postoperative pain compared to general anaesthesia and wound infiltration for major oncological breast surgery

**DOI:** 10.1186/2193-1801-3-517

**Published:** 2014-09-11

**Authors:** Esther A C Bouman, Maurice Theunissen, Alfons GH Kessels, Kristien BMI Keymeulen, Elbert AJ Joosten, Marco AE Marcus, Wolfgang F Buhre, Hans-Fritz Gramke

**Affiliations:** Department of Anaesthesiology and Pain Management, Maastricht University Medical Center, P. Debyelaan 25, 6202 AZ Maastricht, The Netherlands; Department of Clinical Epidemiology and Medical Technology Assessment (KEMTA), Maastricht University Medical Center, Maastricht, The Netherlands; Department of Surgery, Maastricht University Medical Center, Maastricht, The Netherlands; Department of Anaesthesiology, ICU and Perioperative Medicine, HMC, Doha, Qatar

**Keywords:** Paravertebral block, Wound infiltration, Postoperative pain, Breast surgery, Randomized controlled trial

## Abstract

We hypothesized that improved acute postoperative pain relief will be achieved using general anaesthesia (GA) either in combination with continuous thoracic paravertebral block (GA-cPVB) or single shot (GA-sPVB) as compared to GA supplemented by local wound infiltration (GA-LWI) after unilateral major breast cancer surgery.

A randomised controlled trial was conducted in 46 adult women in a day-care or short-stay hospital setting after major breast cancer surgery. Pain-intensity was measured using an 11-point visual analogue scale (VAS) until postoperative day 2. GA-sPVB was stopped due to slow inclusion.

No significant difference in VAS score was noted between GA-LWI (VAS median 0.5 (interquartile range 0.18–2.00)) and GA-cPVB, (VAS 0.3 (0.00–1.55, *p* = 0.195)) 24 hours after surgery or at any point postoperatively until postoperative day 2.

We conclude that both GA-LWI and GA-cPVB anaesthetic techniques are equally effective in treatment of acute postoperative pain after major oncological breast surgery. As GA-LWI is easily to perform with fewer complications and it is more cost-effective it should be preferred over GA-cPVB.

## Introduction

Optimal acute postoperative pain relief after major surgical breast surgery is still a matter of debate. After major oncological breast surgery patients still suffer from acute postoperative pain. Data of our own patient population (Sommer et al. 2008) showed that 22% of the patients reported mean VAS of >40 (of a scale 0–100) on the first postoperative day after major breast surgery. This was confirmed in a recent cohort study with a reported mean pain score of 3.82 (SD 2,47) on the postoperative day 1 (Gerbershagen et al. 2013; Gerbershagen et al. 2014). In this context, a paravertebral block (PVB) which provides a unilateral segmental nerve block is advocated as the technique of choice for breast surgery (PROSPECT working group 2008). Previous studies observed improved acute postoperative pain management (Boughey et al. 2009; Kairaluoma et al. 2004; Klein et al. 2000; Naja et al. 2003; Terheggen et al. 2002), less nausea (Kairaluoma et al. 2004; Klein et al. 2000; Naja et al. 2003), faster recovery from anaesthesia (Kairaluoma et al. 2004), earlier hospital discharge (Naja et al. 2003), and reduced incidence of chronic postoperative pain when PVB is used (Kairaluoma et al. 2006). The lower incidence of postoperative nausea and vomiting (PONV) and the faster recovery makes PVB an attractive analgesic approach to day care surgery, as a significant amount of the surgical procedures are currently performed in this setting (in the Netherlands 51% (Statistics Netherlands (CBS) 2012)). The majority of studies only compared general anaesthesia (GA) with PVB and systemic pain therapy with the use of intravenous opioids. However, in daily practice local wound infiltration (LWI) with local anaesthetics is commonly used complementary to systemic analgesics for postoperative pain relief (Johansson et al. 2003; Sidiropoulou et al. 2008; Vigneau et al. 2011). Therefore, the use of GA with PVB (GA-PVB) should be compared to GA with LWI (GA-LWI).

In this study, the primary objective was to determine analgesic effects of a GA combined with PVB as compared to GA with LWI in patients undergoing major breast surgery in day or short stay hospital setting. We hypothesised that better acute postoperative pain relief 24 hours after surgery (Day 1) could be achieved using GA-PVB as compared to GA-LWI.

## Methods

This study was carried out in compliance with the Helsinki Declaration. Following approval by the medical ethics commitee of Maastricht University Medical Center+ (reference number MEC 05-105), written informed consent and registration at http://www.clinicaltrials.gov identifier: NCT00547989, patients were included in a prospective, open, randomised controlled trial. The study population consisted of adult women scheduled for one-sided, major breast cancer surgery. Surgical procedures included wide local excision (WLI), mastectomy and modified radical mastectomy (MRM). Sentinel node procedure, axillary dissection, or immediate prosthetic breast reconstruction was mandatory in case of WLI and optional in case of mastectomy or MRM.

All patients were ASA class I or II and planned for day care or short stay surgery. Exclusion criteria were as follows: contra-indication for regional anaesthesia, coagulation disorders, infection at point of insertion, infection in thoracic cavity, tumour in paravertebral area, history of pleurectomy, and history of allergic reaction to contrast medium or local anaesthetics.

Patients were randomised in two phases, using a computer generated list. First, patients were assigned to GA plus local wound infiltration (GA-LWI) or GA plus PVB (GA-PVB). Secondly, patients in the PVB group were then randomised either in a subgroup with single shot PVB (GA-sPVB)) or in a subgroup with continuous PVB (GA-cPVB) using a paravertebral catheter and patient controlled analgesia.

Patients in groups GA-sPVB and GA-cPVB received a thoracic paravertebral block preoperatively according to a standard technique described in detail elsewhere (Terheggen et al. 2002; Eason & Wyatt 1979). A member of the study group (either EB or HG) performed all procedures. Briefly, a 20 Gauge catheter (B.Braun Melsungen AG, Melsungen, Germany) was inserted 3 cm into the paravertebral space at thoracic level 3–4, using an 18 Gauge Tuohy cannula needle. After a test dose of 3 ml ropivacaine 0.75%, a total dose of 0.25 ml/kg ropivacaine 0.75% was injected. Postoperatively, the position of the catheter was confirmed by thoracic X-ray and injection of 2–3 ml contrast medium (Iohexol 240 mg l/ml, Omnipaque® GE Healthcare B.V. The Netherlands) via the catheter.

Induction of GA was performed with propofol 2–3 mg/kg and sufentanil 0.1-0.2 μg/kg at induction, rocuronium 0.6 mg/kg to facilitate endotracheal intubation or laryngeal mask airway. Maintenance of anaesthesia was performed according to hospital practice in general with sevoflurane/air (0.9-1.3 MAC) and additional boluses of sufentanil as clinically deemed necessary.

Surgery was performed by or under close supervision of a dedicated staff surgeon.

Patients in group GA-cPVB received continuous infusion of ropivacaine 0.2% at 5 ml/h plus an optional patient controlled bolus of 5 ml (lock-out interval: one hour) (Easypump® RA 400–5 PCA, B. Braun Melsungen AG, Melsungen, Germany). Adjunct postoperative analgesia in all groups consisted of paracetamol (4 × 1000 mg) fixed dose and a non-steroidal anti-inflammatory drug (NSAID) (naproxen or diclofenac) in combination with piritramide and ondansetron as required. Day care patients were allowed to stay overnight in case of delayed recovery.

Patient baseline characteristics age, length, weight, ASA classification, and surgical data were recorded. At the PACU, vital signs on arrival and nausea (Numeric Rating Scale, NRS 0–10) were registered. The primary outcome measure, postoperative pain, was measured using a visual analogue scale ranging from 0 to 10 (VAS). A pain score of less than 4 on the VAS was considered as sufficient for postoperative analgesia (Jensen et al. 2003). Postoperative pain was measured on arrival at the PACU, on discharge from the PACU, and from then on three times per day (at 8.00-14.00-20.00 hr.) for two postoperative days. After discharge from the PACU the patients used a pain diary to record the pain scores. In addition, patients were asked to report the use of analgesics as well as the overall satisfaction with pain treatment (5-point-verbal rating scale). Initially a GA-sPVB patient group was planned and included in which the paravertebral catheter was removed at the PACU after an additional dose of 10 ml ropivacaine 0.2%. However, due to very low inclusion rate of patients in the GA-sPVB group the Medical Ethical Committee suggested to stop the inclusion of patients into this group.

Patients in group GA-LWI received local wound infiltration with10 ml bupivacaine 0.25% before wound closure according to the standard procedure for extended mamma surgery as used in our hospital (MUMC+, the Netherlands).

In order to o detect a 1.5 (SD 1.5) VAS pain score difference at 24 hours after surgery (Kairaluoma et al. 2004) with a power of 80% and a significance level of 5%, our analysis revealed that 16 patients needed to be included per group. Assuming a drop-out of 10%, we decided to include 18 patients per group.

Baseline data and secondary outcomes were analysed using student’s *t*-test and Fisher Exact Tests for parametric data, Mann Whitney U-tests for non-parametric data, and Chi-square tests for categorical data. Multivariate analysis of the primary VAS score outcome was performed using a multilevel linear model. Differences between GA-LWI and GA-cPVB as a function of time were assessed with the intervention and time as fixed effects. Interaction between treatment group and time was assessed.

Adjustment for covariates, age, ASA-classification, perioperative opioid use, and type of surgery was entered in the model. For the primary outcome measure, substitution of missing data was not performed as the multilevel linear model is sufficiently robust in handling missing data.

All data were analysed according to the intention to treat principle, using the Statistical Package for the Social Sciences (SPSS® version 18, Chicago, Illinois, USA). A *p* value < 0.05 was considered statistically significant.

## Results

**Figure 1:**
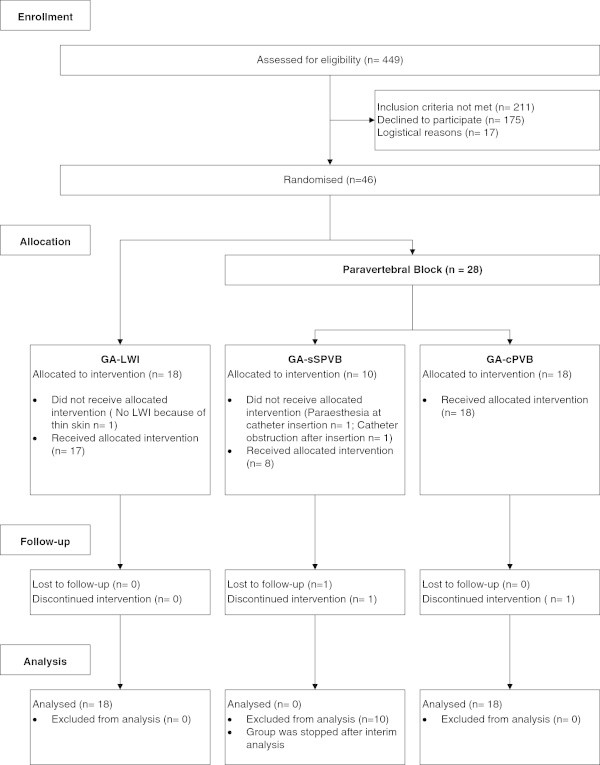
**Consort flow chart.** GA-LWI: general anaesthesia and local wound infiltration, GA-sPVB: general anaesthesia and single shot paravertebral block, GA-cPVB: general anaesthesia and continuous PVB.

Trial recruitment was scheduled from October 2006 to April 2011. A total of 449 patients were screened. The proportion of eligible patients was 53% (238) of whom 19% (46) gave informed consent (Figure 1). An interim analysis was performed in October 2009. Due to low inclusion rate it was decided to exclude the GA-sPVB group from analysis. The present analysis is therefore based on a total of 36 patients: GA-cPVB (n = 18) and GA-LWI (n = 18). No relevant significant differences were noted between groups with regard to baseline characteristics or type of surgery (Table 1). Patients in the GA-LWI group received significantly more opioids intraoperative than patients in the GA-cPVB group (Table 1).

**Table 1:** **Population characteristics and intraoperative data**

	GA-LWI n = 18	GA-cPVB n = 18	***p***value
Age (years)	57.9 (13.8)	60.9 (12.7)	0.52
Length (cm)	166.7 (6.9)	165.6 (4.2)	0.55
Weight (kg)	70.8 (17.5)	67.4 (9.43)	0.47
ASA I/II	9 / 9	5 / 13	0.17
Airway TT/LMA	11/7	10/8	0.74
Total dose sufentanil (μg)	33.1(10.6)	25.1(9.5)	0.024
Intraoperative infusion (ml)	1194 (300)	1428(379)	0.048
Duration of anaesthesia (hours)	2.50(0.69)	2.57(0.66)	0.78
Duration of surgery (hours)	1.98(0.61)	2.09(0.58)	0.58
Type of surgery			0.25
Lumpectomy/Ablatio/MRM +/− sentinel node	12	7	
Ablatio/MRM + Axillary node	3	6	
Ablatio/MRM + Plastic surgery +/− Axillary node	3	5	

GA-LWI: general anaesthesia and local wound infiltration, GA-cPVB general anaesthesia and continuous PVB, TT: tracheal tube; LMA: laryngeal mask airway.

MRM: modified radical mastectomy. Values are numbers or mean (SD).

**Figure 2:**
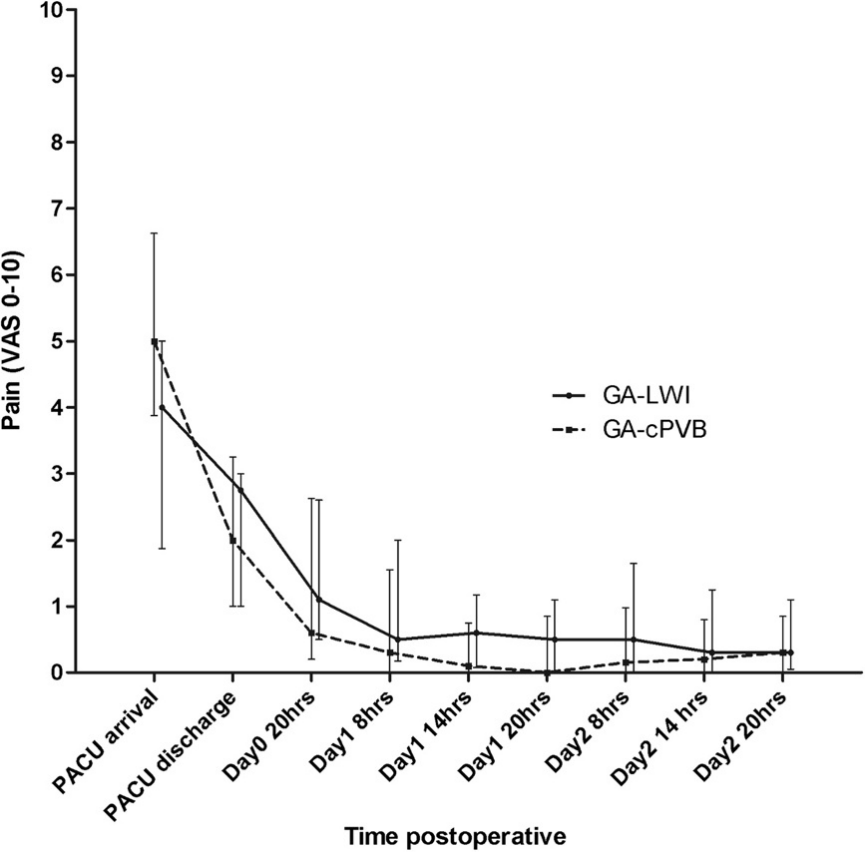
**Postoperative pain scores.** Values are VAS pain, median IQR. PACU: post anaesthetic care unit.

There was no significant difference in the primary outcome parameter VAS score between GA-LWI (VAS median 0.5 (0.18–2.00)) and GA-cPVB, (VAS median 0.3 (0.00–1.55, *p* = 0.195)) 24 hours after surgery. No difference in VAS score between GA-LWI and GA-cPVB was noted at any time point postoperatively until postoperative day 2 (Figure 2).

Moreover, no significant differences between GA-LWI and GA-cPVB were noted in the number of patients that used opioids on the day of surgery, although the amount of opioids used was significantly lower in GA-cPVB as compared to GA-LWI (Table 2). This difference in the amount of opioids per patient was not associated with a difference in the incidence of postoperative nausea or the need for anti-emetic drugs in the PACU. Patient satisfaction questionnaire revealed equal results in GA-LWI and GA-cPVB (Table 2).

**Table 2:** **PONV, postoperative opioid use and satisfaction with analgesic treatment**

	GA-LWI n = 18	GA-cPVB n = 18	***p***value
PACU Nausea at discharge	0.9(1.4)	1.0(1.5)	0.82
PACU anti-emetic use (yes/no/missing)	13/4/1	14/4/0	1.00
Opioid use day 0 (yes/no/missing)	7/10/1	6/12/0	0.63
Piritramide day 0 (mg)	7.5 (0–26)	1.5 (0–8)	0.03
Satisfaction with treatment			0.87
Bad - Moderate	0	1	
Good	2	2	
Very good - Excellent	13	14	
missing	3	1	

GA-LWI: general anaesthesia and local wound infiltration; GA-cPVB: general anaesthesia and continuous paravertebral block. Values are numbers, mean (SD), or mean (range).

The placement of the paravertebral catheter was successful in all patients. A vascular puncture occurred in one patient. The median indwelling time from start of surgery was 43.3 (IQR 41.7-46.3) hours.

Analysis of the course of pain on postoperative Days 1 and 2 using the multilevel linear model with VAS pain score as a function of time (hours) as the dependent variable revealed that the effect on postoperative pain did not differ between GA-cPVB and GA-LWI.

The postoperative time course (hours) and the interaction between intervention and time were significant predictors of postoperative VAS-pain score in both GA-LWI and GA-cPVB (Table 3).

**Table 3:** **Multilevel analysis parameter estimates of fixed effects**

Parameter	Estimate	95% CI		***p***value
		Lower Bound	Upper Bound	
GA-cPVB	−0.283	−1.287	0.721	0.57
Time after surgery	−0.052	−0.064	−0.039	0.000
GA-cPVB* Time	−0.023	−0.041	−0.005	0.012
Age	0.081	−0.027	0.043	0.64
ASA	−0.223	−1.073	0.628	0.60
Additional axillary dissection	0.184	−0.847	1.215	0.72
Additional immediate prosthetic breast reconstruction	0.715	−0.522	1.952	0.25
Total dose sufentanil	0.130	−0.030	0.056	0.54

GA-cPVB: general anaesthesia and continuous paravertebral block, cPVB * Time: interaction effect between intervention and time after surgery.

**Figure 3:**
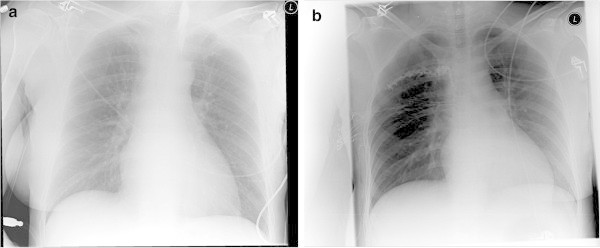
**Examples radiographic contrast medium spread in patients with continuous paravertebral block. a**. radiographic contrast medium spread in thoracic paravertebral space. **b**. Intercostal spread of radiographic contrast medium.

Initially, VAS-pain score decreased rapidly; thereafter a slight decrease in pain intensity was observed which lasted several hours, for both groups (Figure 2). The average reduction of 0.5 on VAS at postoperative day 1 is attributed to continuous paravertebral block using PCA. Assessment of potential confounding factors like age, ASA classification, and type of surgery did not reveal significant effects (Table 1).Thoracic X-ray confirmed the correct position of the paravertebral catheter and revealed no pneumothorax. The contrast medium mainly spread along the thoracic paravertebral space (n = 11) a cloud like pattern (n = 3) an intercostal spread (n = 2) or a combination of paravertebral and intercostal spread (n = 2) (Figure 3).

We observed only minor complications after GA-cPVB: minor bleeding at the puncture site in one patient, premature catheter dislocation in another patient. Furthermore, three patients complained about back pain, and one patient was not capable of pushing the PCA button.

## Discussion

The results of the present study suggest that general anaesthesia combined with local wound infiltration (GA-LWI) and continuous paravertebral infusion of local anaesthetics (GA-cPVB) are equally effective in acute postoperative pain relief. Patients receiving GA-cPVB used a significantly lower amount of systemic opioids as compared to patients in GA-LWI group. The increased amount of systemic opioids was, however, not associated with an increased incidence of postoperative PONV in group GA-LWI.

In the present study, correct placement of the paravertebral catheter was confirmed via chest X-Ray after surgery. Furthermore, PVB was performed by two members of the study team (EB, HG), in a standardised manner that contributed to the high success rate of the technique. A basic analgesic regimen was prescribed for all patients and surgery was performed by or under close supervision of a dedicated staff surgeon.

The main limitation of our study was the slow inclusion rate and a low proportion of screened patients suitable for randomisation, as earlier reported (Buckenmaier et al. 2010). Another limitation might be the fact that the study was not performed in a blinded fashion. In this context it is important to note that due to the potentials risk of the PVB procedure and the eventual burden for study participants, sham PVB was not considered an option.

Relatively low pain scores were noted in both GA-LWI and GA-cPVB groups on postoperative day 1. These low pain scores, in particular in the GA-LWI group, were not expected as a previous study of our group (Sommer et al. 2008) reported 22% of the patients having a mean VAS at rest >40 (scale 0–100) on the first postoperative day (Sommer et al. 2008).

The combination of assistance of an oncological support team (mamma-care nurse), a dedicated study team and a motivated patient group may have been contributed to these low pain scores at postoperative day 1. Moreover all patients received a basic analgesic regimen including paracetamol (4 × 1000 mg) fixed dose and a non-steroidal anti-inflammatory drug (NSAID) (naproxen or diclofenac) in combination with piritramide and ondansetron as required.

Median and worst pain scores in patients undergoing different surgical procedures were analysed recently in two cohort studies (Gerbershagen et al. 2013; Gerbershagen et al. 2014). In the subgroup of patients undergoing breast surgery, mean numeric rating scale (NRS) was 3.26 and 2.98 for major and minor breast surgery, respectively. These cohort studies did not present detailed information on the use of PVB or LWI nor is there information provided on the pain scores 24 hours after surgery (Gerbershagen et al. 2013; Gerbershagen et al. 2014). Nevertheless, we conclude that patients in our study showed postoperative VAS scores comparable to those reported for the general population undergoing this type of surgery. Moreover, three RCTs addressed the same issues. Continuous wound infiltration was compared with single injection paravertebral block and low absolute postoperative VAS-pain scores in both groups up to 24 hours after surgery was reported (Sidiropoulou et al. 2008). These results are in line with the present study where absolute postoperative VAS-pain scores in both GA-cPVB and GA-LWI groups were low and were comparable between the groups. It should be stressed that in the study of Sidiropoulou (Sidiropoulou et al. 2008) both groups received more systemic opioids after surgery than patients from the present study. Systemic opioid use is commonly associated with an increased incidence of nausea and vomiting (Miaskowski 2009) a finding that, however, cannot be supported by the results of the present study.

The results of the present study and those presented by Sidiropoulou (Sidiropoulou et al. 2008) differ from a number of studies in which GA alone and GA-PVB were compared. For instance, no difference between groups was found when effects of single shot PVB alone, combined with continuous PVB and placebo was studied in 74 patients undergoing breast surgery (Buckenmaier et al. 2010). The latter study includes, however, some major limitations. Not only the PVB technique used was a mixture of a single shot and a multilevel approach, also the assignment of patients to the 3 groups was stratified by surgery class, and patients were deeply sedated during the procedure and conversion to GA was necessary in 12% of the patients. Furthermore 21 patients with incomplete data were excluded from the analysis (Buckenmaier et al. 2010).

Most recently, an interesting randomised controlled study (Abdallah et al. 2014) was published which compared multilevel PVB/total intravenous anaesthesia (TIVA) and a balanced volatile anaesthetic technique. Here lower pain scores and improved recovery scores were reported in the PVB/TIVA group as well as a reduced incidence of nausea and vomiting. Comparison with the data from our study is difficult as study designs considerably differ. In the study of Abdallah (Abdallah et al. 2014) patients in the volatile general anaesthesia group also received nitrous oxide, whereas patients in the TIVA group received propofol and oxygen in air. Both, volatile anaesthetics and nitrous oxide can contribute to the increased incidence of nausea and vomiting and can, at least in part, explain the lower recovery scores in the general volatile anaesthesia group as noted in this study (Abdallah et al. 2014). Even more important, the patients received no local wound infiltration and no basic analgesic regimen. Interestingly, median pain scores in the PVB group (Abdallah et al. 2014) were comparable to the results of the present study when patients met the discharge criteria from the PACU, suggesting that LWI, cPVB and multilevel single shot PVB are comparable with respect to peri-operative pain scores.

In a large observational single institution study patients with breast surgery not undergoing early reconstructive surgery, no differences in nausea, vomiting and postoperative pain scores were observed (Aufforth et al. 2012). However hospital charts of patients were retrospectively analysed.

The efficacy and safety of paravertebral blocks in breast surgery was calculated based on a meta-analysis of randomised clinical trials (Schnabel et al. 2010). In this meta-analysis, 15 RCTs were included with a total of 877 patients. Then significant differences in pain scores in the initial period (<2 h) as well as up to 48 hours for both the combination of PVB and general anaesthesia vs. GA alone were reported (Schnabel et al. 2010). The observation that the funnel plot showed asymmetry might be of significant importance and suggests publications bias regarding negative study results.

Although the direct postoperative VAS scores at PACU arrival in our study were somewhat higher, we noted that VAS values dropped consistently in both GA-cPVB and GA-LWI groups. From this we may conclude that LWI is a cost effective and low risk procedure and seems to be comparable to the PVB approach.

The results of our study are encouraging as LWI is easy, readily available and has almost no side effects. It is less invasive than other regional techniques like paravertebral and interpleural blocks (Kundra et al. 2013) and there is no need for any technical device or follow up for catheter removal. From our study we tentatively conclude that in contrast with more painful procedures the wearing off of local anaesthetic effect after wound infiltration is not a major determinant for pain during the next days.

We have demonstrated that both GA-LWI and GA-cPVB techniques were effective in treatment of acute postoperative pain after major oncological breast surgery. As GA-LWI is easily to perform with fewer complications and it is more cost-effective it should be preferred over GA-cPVB. A possible additional value of continuous paravertebral block in more painful extended procedures has to be investigated.
